# Supplementary material for: High-fidelity, efficient, and reversible labeling of endogenous proteins using CRISPR-based designer exon insertion
Source: eLife. 2021 Jun 8;10:e64911. doi: 10.7554/eLife.64911 (PMC8211447; doi:10.7554/eLife.64911)
Supplement: Supplementary file 1. [file elife-64911-supp1.docx]

| Primer # | Sequence |
| --- | --- |
| P#1 | CCGCGAAGCCGGCCTTGCAC |
| P#2 | GAGCACAGAGCCTCGCCTTTGC |
| P#3 | GAGCCTCGCCTTTGCCGATCCG |
| P#4 | GCCAGATTTTCTCCATGTCGTCCC |
| P#5 | CACCATCACGCCCTGGTGCC |
| P#6 | GACCCGGCGCTGTTTGAACC |
| P#7 | GCTGTTTGAACCGGGCGGAGG |
| P#8 | CATCGTCGCCCGCGAAGCC |
| P#9 | GGTGCATTAAGGGTATTGCCTTAG |
| P#10 | GTGAGGGTGGAGGACCTGTAC |
| P#11 | GTTTACGTCGCCGTCCAGCTC |
| P#12 | CACCACCCCGGTGAACAGCTC |
| P#13 | GCGGACTTGAAGAAGTCGTGC |
| P#14 | GCTTCATGTGGTCGGGGTAGC |
| P#15 | CCTGCTGGAGTTCGTGACCG |
| P#16 | CGAGAAGCGCGATCACATGG |
| P#17 | CTGCTCCTGTCGCCTTCG |
| P#18 | CGCCTCCTAATCCCTAGCCAC |
| P#19 | GACTGCGGGGACTCGAGG |
| P#20 | CCCAGGACACAGTTACGCG |
| P#21 | GTGTAGTGCCCTCGGGCATAG |
| P#22 | GCAGCATCTTCCTTGCCTGTG |

**Supplementary Table 1. PCR primer list.**
