# Supplementary material for: High-fidelity, efficient, and reversible labeling of endogenous proteins using CRISPR-based designer exon insertion
Source: eLife. 2021 Jun 8;10:e64911. doi: 10.7554/eLife.64911 (PMC8211447; doi:10.7554/eLife.64911)
Supplement: Supplementary file 2. [file elife-64911-supp2.docx]

|  |  | First round primers | | Second round (nested) primers | | Expected size (bp) |
| --- | --- | --- | --- | --- | --- | --- |
| *ACTB* Genomic DNA | No donor insertion | P#6 | P#8 | P#7 | P#1 | 325 |
|  | Donor insertion, forward, 5' | P#6 | P#11 | P#7 | P#12 | 314 |
|  | Donor insertion, forward, 3' | P#9 | P#8 | P#10 | P#1 | 324 |
|  | Donor insertion, inverse, 5' | P#6 | P#9 | P#7 | P#10 | 179 |
|  | Donor insertion, inverse, 3' | P#11 | P#8 | P#12 | P#1 | 459 |
|  |  |  |  |  |  |  |
| *ACTB* mRNA | No donor insertion | P#2 | P#4 | P#3 | P#5 | 187 |
|  | Donor insertion, forward, 5' | P#2 | P#13 | P#3 | P#14 | 308 |
|  | Donor insertion, forward, 3' | P#16 | P#4 | P#15 | P#5 | 247 |
|  | Donor insertion, inverse, 5' | P#2 | P#16 | P#3 | P#15 | n/a |
|  | Donor insertion, inverse, 3' | P#13 | P#4 | P#14 | P#5 | n/a |
|  |  |  |  |  |  |  |
| *TUBA1B* | Genomic DNA, ctrl | P#17 | P#19 | P#18 | P#20 | 324 |
|  | mRNA, ctrl | P#17 | P#21 | P#18 | P#22 | 321 |

**Supplementary Table 2. PCR primer combinations.**
