# Supplementary material for: High-fidelity, efficient, and reversible labeling of endogenous proteins using CRISPR-based designer exon insertion
Source: eLife. 2021 Jun 8;10:e64911. doi: 10.7554/eLife.64911 (PMC8211447; doi:10.7554/eLife.64911)
Supplement: Supplementary file 3. [file elife-64911-supp3.docx]

This protocol provides a workflow and step-by-step instruction for the CRISPIE method using *ACTB* labeling as an example.

1) Identifying the potential targeting site.

a. Based on literature review, EGFP-β-actin has been successfully used in overexpression conditions, suggesting that it may be possible to label actin at its N terminus.

b. Download and examine the ACTB gene structure from the NCBI website (<https://www.ncbi.nlm.nih.gov/gene/>).

c. By examining the gene structure, intron 1 appears to be an appropriate location for tagging because the start codon of ACTB is located within a few bps from the beginning of exon 2 (see also Figure 1C).

d. The sequence of intron 1 is copied and pasted to the GPP sgRNA designer hosted by the BROAD institute (<https://portals.broadinstitute.org/gpp/public/analysis-tools/sgrna-design>) to identify candidate sgRNA targeting sites. We suggest testing at least 3 sites, including one near the upstream exon and one near the downstream exon (within 500 bp), as their efficiency may vary (Figure 3B). At the same time, try to avoid sites that are within 100 bp of intron-exon junctions because they may potentially affect splicing.

2) Making/identifying the constructs

a. Order both forward and inverse oligos containing the target-specific sgRNA sequence. For example, for Figure 1, the forward primer CACCGaaaaggcaaacactggtcgg and reverse primer AAACccgaccagtgtttgccttttC were ordered. The target-specific sequence is underlined. Other sequences are ligation-compatible overhangs (purple) and a G (blue) that is required for RNA transcription initiation using the U6 promotor.

b. Anneal and ligate into pX330 (<https://www.addgene.org/42230/>) cut by the restriction enzyme BbsI-HF. This vector also expresses SpCas9 in addition to the sgRNA.

c. For initial testing, identify a generic donor vector of the appropriate reading frame (phase) that expresses the appropriate FP. The generic donor vector also expresses the sgRNA needed to excise the donor sequence (e.g., Figure 1–figure supplement 3B6 – 3B10). For example, intron 1 of *ACTB* is phase 0. This means that the first three nucleotides of the next exon (exon 2) will code an amino acid residue. B7 in Figure 1–figure supplement 3B can therefore be used to tag *ACTB* with mEGFP.

d. (Optional) For higher labeling efficiency, one may consider constructing a target-specific donor similar to that in Figure 1–figure supplement 3B1. This can be done by ordering the appropriate oligos containing the sgRNA targeting sequence and the appropriate restriction enzyme sequences, and using them to replace the previous sgRNA targeting sequence in an existing donor vector. All plasmids in this manuscript will be made available at Addgene. The sgRNA targeting sequence should be in the reverse orientation compared to the genomic sequence. This allows the donor sequence to be preferentially inserted in the correct orientation. We empirically find that target-specific donors give a 50 – 100% higher labeling efficiency compared to generic donors.

3) Labeling the endogenous protein.

a. Mix the appropriate amount of the sgRNA/SpCas9 plasmid with the donor plasmid. We typically use 0.5 µg and 1 µg, respectively, of DNAs for a 35 cm tissue culture dish. The exact amount may vary depending on the transfection method and cell type.

b. Add a small amount of plasmid of a transfection marker. We typically use 0.2 µg of mRuby3 for green CRISPIE labeling. Most other red FPs aggregates or exhibit a significant green component (or bleed-through), making it difficult to know whether the targeted protein is labeled (green). Note that the labeling of the majority of proteins at endogenous levels will be dim. mRuby3 also has a low level of green component, so only a small amount of the plasmid should be used.

c. Follow the manufacturer’s protocol of lipofectamine 2000 (ThermoFisher) to transfect cells with the exception below. For actin labeling, we use U2OS cells, which are passed the day before and are at 30-50% confluency at transfection. We typically use 5 µl lipofectamine 2000 per well for a 35 cm dish, which is half of the manufacturer’s recommended amount. Specifically for U2OS cells, the medium will be replaced after 5.5 hours, and once more at 6 hours after transfection, to reduce toxicity resulted from the transfection reagent.

d. The cells will be examined 3 days after transfection under an upright microscope. For experiments involving inverted microscopes, the cells will be re-plated into coverslip-bottomed dishes (WPI #FD35-100) or 6 well plates (Cellvis #P06-1.5H-N), and imaged 0–3 days after re-plating depending on the experiment.

e. Other transfection methods should also work. For biolistic transfection, we typically use 3 µg sgRNA/SpCas9, 6 µg donor, and 0.3 µg mRuby3 plasmids per half-a-tube bullet preparation (Bio-Rad). Transfected cultured hippocampal slices are imaged 7–10 days post transfection. For in utero electroporation, we typically use ~1.2 µg sgRNA/SpCas9, 1.8 µg donor, and 0.8 µg mRuby3 (endotoxin-free maxiprep DNA) in one microliter per embryo. Electroporation is typically carried out at E15.5 for layer 2/3 cortical neurons. We typically examine in acute slices at p20 – p30, or install cranial window for *in vivo* imaging at ~p45 – p60.
